# Foot-related/walking macro-affordances are implicitly activated and preferentially guided by the framing distance of the environmental layout

**DOI:** 10.1007/s00426-022-01692-w

**Published:** 2022-07-06

**Authors:** Annalisa Tosoni, Emanuele Cosimo Altomare, Mauro Gianni Perrucci, Giorgia Committeri, Rosalia Di Matteo

**Affiliations:** 1 Department of Neuroscience, Imaging and Clinical Sciences, Gabriele D’Annunzio University of Chieti-Pescara, Via Luigi Polacchi 11, 66100 Chieti, Italy; 2 Institute for Advanced Biomedical Technologies (ITAB), Gabriele D’Annunzio University of Chieti-Pescara, Chieti, Italy

## Abstract

As classically captured in the notion of affordance, the natural environment presents animals with multiple opportunities for action and locomotion appears as the privileged form of action to cover distance in the extrapersonal space/environment. We have recently described a facilitation effect, known as “macro-affordance”, for the execution of walking-related actions in response to distant vs. near objects/locations in the extrapersonal space. However, since the manipulation of distance was coextensive to landmark-objects contained in the environment and to the environmental layout per se, the relative contribution of these two factors remains undetermined. In addition, since the effect was originally described in the context of an incidental priming paradigm, it is still unknown whether it was specifically associated with an implicit coding of environmental distance. Here, across three experiments, we examined the degree to which the “macro-affordance” effect reflects (i) the encoding of environmental vs. landmark-objects’ distance, (ii) the involvement of an implicit vs. controlled system, (iii) a foot-effector specificity. The results showed that the “macro-affordance” effect is more efficiently triggered by the framing distance of the environmental layout (far/wide/panoramic vs. near/close/restricted) rather than of isolated landmark-objects in the environment and that it only emerges when the distance dimension is implicitly processed within the incidental priming paradigm. The results additionally suggested a specificity of the effect for foot- vs. hand-related actions. The present findings suggest that macro-affordances reflect an implicit coding of spatial features of the environmental layout and viewer–environment relationships that preferentially guide a walking-related exploration of the spatial environment.

## Introduction

Animals’ survival is likely guaranteed by a backbone of neural mechanisms developed to support adaptive interactions between animals and their extended natural environment. Within such evolutionary perspective, the brain would have evolved to satisfy the request of a constantly changing environment that presents animals with multiple opportunities and demands for actions.

At the theoretical level, such an intimate and mutual relationship between animals and their surrounding environment has been originally conceptualized in the notion of affordance (Gibson, 1979), defined as a property of the environment providing the viewer with practical opportunities of action, and successively further refined and expanded by embodied and ecological views of perception (Creem-Regehr & Kunz, 2010).

At the experimental level, the notion of affordance has been principally investigated in the realm of the interaction between hand-related actions and manipulable objects/tools. For example, using an incidental object categorization task, such as judging whether an object was upright vs. inverted or natural vs. manufactured, a response-compatibility effect was described in the seminal studies by Tucker and Ellis for the execution of functionally appropriate hand-related actions to visually presented objects (e.g., spatial alignment between the responding hand and the handled object). This effect of motor potentiation, known as “micro-affordance”, refers to a decrease of reaction times for components of hand-related actions afforded by seen objects (Ellis & Tucker, 2000; Tucker & Ellis, 1998, 2001). Crucially, since this motor potentiation or compatibility effect was typically observed in the context of incidental tasks not requiring participants to explicitly pay attention to the affording properties of the presented objects (e.g., upright vs. inverted object orientation), the micro-affordance effect has been typically considered an automatic mechanism, i.e., an automatic activation of representations of actions during object/tool perception.

Converging evidence from experimental work, however, has demonstrated that the micro-affordance effect is rarely or not observed at all during tasks requiring superficial processing of the object perceptual properties, such as during color judgment tasks, or during conditions in which the object is located in the “out of reach” portion of space (Costantini et al., 2010, 2011; Pellicano et al., 2010; Tipper et al., 2006), leading to the hypothesis that affordances activation is flexibly modulated by the task and by the physical and social context [(i.e., both automatic and task-dependent affordances activation, see (Borghi & Riggio, 2015) for a full discussion].

As noticed above, however, the specific focus on the hand–object relationship might have limited, or somehow circumscribed, the discussion on affording properties of objects in the external world and the mechanism of affordance in general. This issue becomes even more critical when considering the emphasis, in the original formulation of affordance, on natural behavior and adaptive interactions between animals and their extended natural environment.

On this basis, a hypothesis was formulated in our previous work (Di Marco et al., 2019) about a potential affordance relationship between the spatial features of the extrapersonal environment and locomotion, a behavior that is essential for survival and ubiquitous among mobile organisms. Specifically, since locomotion appears as the privileged form of action to cover distance and retrieve information contained in the distant environmental/extrapersonal space, a facilitation effect was hypothesized for walking-related actions to distant landmark-objects/locations in the extrapersonal space. More specifically, an affordance effect was hypothesized between the locomotion action and the distant extrapersonal environment, and this effect was expected to take the form of faster initiation times (i.e., release times) for execution of a footstep action, taken as a proxy of walking, in response to far vs. near extrapersonal landmark-objects/locations. To test this hypothesis, a go/no-go incidental priming paradigm was employed requiring the planning/execution of a foot-related action (i.e., simple release of a foot-pedal response button or simple release followed by a footstep ahead) in response to repeated presentations of pictures of an environmental layout containing objects positioned at different distances (far vs. near) from the observer. Consistent with our predictions, a facilitation effect was observed for execution of the footstep action in response to distant (vs. near) landmark-objects/locations in the extrapersonal space and this effect has been referred to as “macro-affordance” to reflect the parallelism with the “micro-affordance” effect observed during the execution of functionally appropriate hand-related actions towards manipulable objects (Altomare et al., 2021; Di Marco et al., 2019; Tosoni et al., 2021).

However, since in this study the manipulation of distance was achieved by moving the camera at different extrapersonal distances along a vector connecting the camera (i.e., point of view) with the objects contained in the environment, the degree to which the macro-affordance effect was guided by the object position (far vs. near) vs. the framing distance of the environmental layout (far/panoramic vs. near/restricted view) could not be determined. Therefore, two (non-mutually exclusive) hypotheses were formulated: one that strongly reflects the analogy with the micro-affordance effect as motor attributes directly included in the representation of visual objects (Tucker & Ellis, 1998) and strictly dependent on the object position/distance, and the other that more generally considers the spatial properties of the general environment and the Gibsonian notion of environmental affordances. Accordingly, while a first basic interpretation of the effect reflects the notion of “object reachability” (i.e., when participants anticipate walking further their action starts faster, “object reachability” hypothesis), a more intriguing interpretation of the effect was also proposed according to which wide and panoramic views of the environmental layout automatically activate mechanisms for walking-related exploration of the distant spatial environment (i.e., “spatial exploration” hypothesis).

In addition, since our macro-affordance effect was observed in the context of a priming paradigm based on an incidental go/no-go task (i.e., go when the target picture is identical to the prime picture), it was unclear whether it was specifically associated with an implicit coding of environmental distance for walking-related actions or whether it could be also observed during a task requiring an explicit distance categorization. Lastly, although a specificity of the macro-affordance effect was originally observed (Di Marco et al., 2019) for motor actions that mimic the initiation of a walking, the degree to which the effect was specifically associated with a particular response type/effector, e.g., foot- vs. hand-related motor actions, has remained an open question from our previous investigations.

Here, three studies are presented that directly address the questions that have remained open from our original study (Di Marco et al., 2019): (i) whether the macro-affordance effect more strongly reflects the encoding of the spatial position/distance of objects in the environment or the encoding of distance of the environment itself, (ii) the degree to which it is associated with the activation of a system operating at an implicit vs. controlled level of information processing, (iii) the degree to which it reflects a specificity for a particular response type/effector (e.g., foot- vs. hand-related motor actions).

## Experiment 1

Experiment 1 addressed the question of whether the macro-affordance effect was preferentially guided by the framing distance of the general environment vs. the distance of isolated landmark-objects (i.e., beach umbrella). As outlined in the Introduction section, a stronger effect of distance was hypothesized for the layout vs. the landmark-object session within the framework of the “spatial exploration” hypothesis, while a stronger modulation of distance was expected for the landmark-object session within the framework of the “object reachability” hypothesis. Since the macro-affordance effect was interpreted on the basis of an automatic activation of mechanisms for spatial exploration/navigation of the environment in our previous studies (Altomare et al., 2021; Di Marco et al., 2019; Tosoni et al., 2021), a stronger effect of distance was predicted for the layout compared to the landmark-object session. In addition, a stronger effect of distance (either in the layout or in the landmark-object session) was hypothesized for footstep actions that mimic the initiation of a walking (vs. simple release) based on our original findings (Di Marco et al., 2019).

### Methods

#### Participants

Twenty-one right-footed, healthy participants, recruited from students of the “G. D’Annunzio” University of Chieti Pescara (14 females, age range: 25.7 ± 5.2 years), were enrolled for experiment 1. Exclusion criteria were applied for pathological conditions and left-foot dominance.

All participants had normal or corrected-to-normal vision, were naïve as to the purposes of the experiments and agreed to participate in the studies without any compensation fee or credit. Written informed consent was obtained for all participants before study participation in accordance with the ethical standards of the 1964 Declaration of Helsinki. The whole study (i.e., experiments 1–3) was approved by the Ethics Committee of G. D’Annunzio University of Chieti, Italy (protocol code 1755 approved on 25 July 2019).

Sample size was defined based on a power analysis (G*Power 3.1.9.2, α error probability = 0.05, power = 0.95) conducted on the effect size associated with the macro-affordance effect obtained in our previous studies [Cohen’s d values ranging between 0.63 and 0.67 in (Altomare et al., 2021; Di Marco et al., 2019)]. A sample size ranging between 18 and 21 participants was obtained in this analysis, which is consistent with the collected sample size.

#### Apparatus and stimuli

**Fig. 1:**
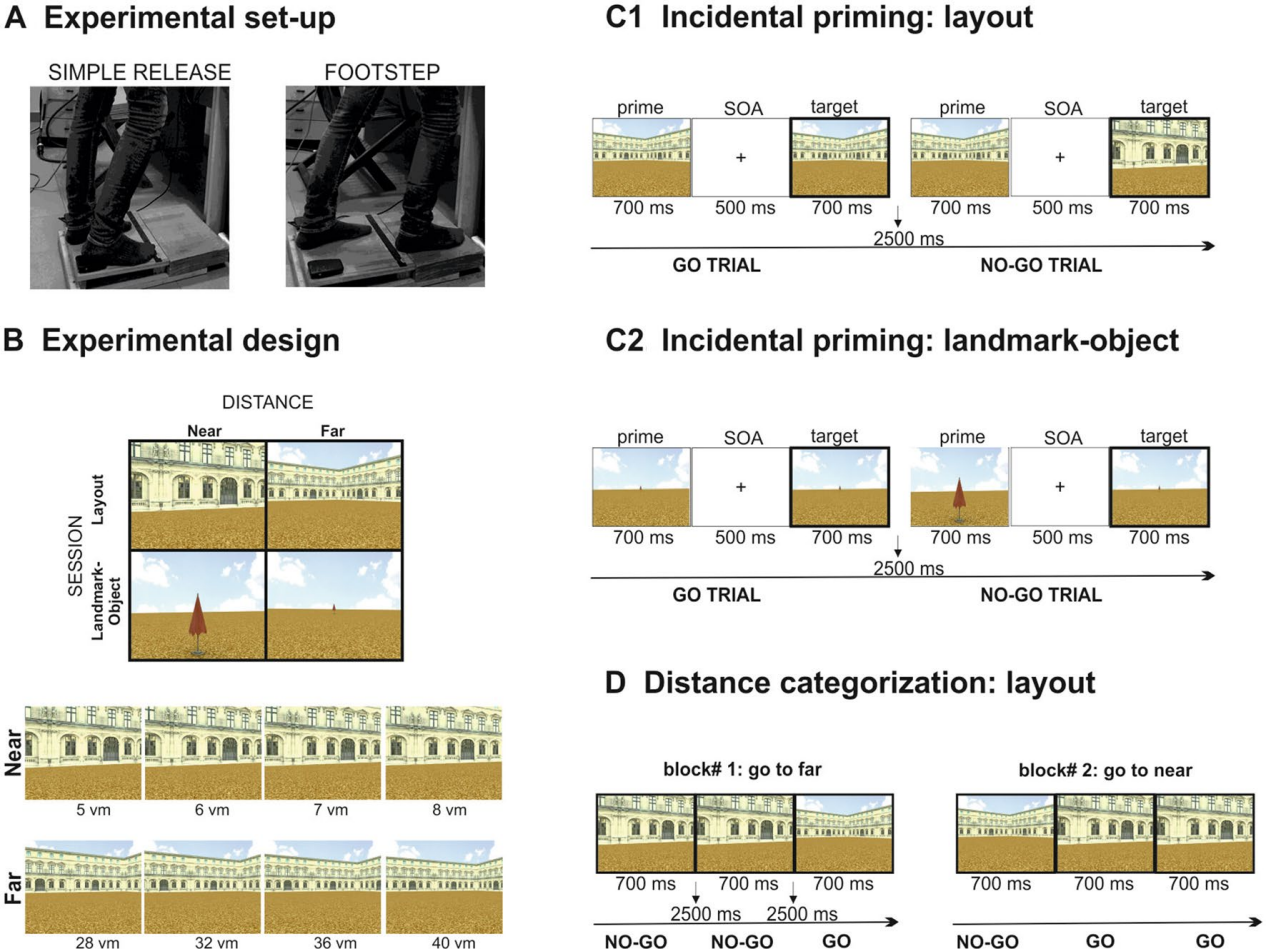
Experimental set-up and design. **A** Set up: Participants were standing in front of a screen covering about 70° of visual angle while holding down a pedal of a foot-related response system with their right heel. **B** Experimental design: Stimuli included a selection of pictures of a 3D virtual environment representing a square arena of a three-winged palace (layout) or a beach umbrella on the same arena (landmark-object) photographed from different extrapersonal distances from the observer (5, 6, 7 and 8 virtual meters for the near condition and 28, 32, 36 and 40 virtual meters for the far condition). **C** Incidental priming paradigm: priming paradigm based on an incidental go/no-go task involving the presentation of pictures pairs (i.e., prime and target) and requiring subjects to execute a footstep ahead or a simple release action based on the prime-target perceptual match, i.e., go trials for perceptually matching pictures. Each picture was presented for 700 ms with an ISI of 500 ms between the two pictures. An ITI of 2500 ms was presented between every pictures pair. **D** Distance categorization task: go/no-go tasks requiring the execution of a previously instructed movement (i.e., footstep, simple release) in response to single pictures of the 3D virtual environment representing a square arena of a three-winged palace (layout) framed from far or near distance. The association between the response movement and the condition of distance was alternated across blocks (i.e., blocks with a go signal for the near condition and a no-go signal for the far condition were alternated with blocks with the opposite association). Each picture was presented for 500 ms with an ITI of 2500 ms between each trial

The experiment was conducted using a set-up consisting of a 42″ widescreen and a foot-pedal response system (BrainTrends ltd., Italy) positioned on the floor at a distance of 57 cm from the screen (Fig. 1A). The experimental stimuli (pictures) were projected on the screen to cover about 70° of visual angle. Stimulus presentation was controlled by E-Prime 2 (Psychology Software Tools Inc., Pittsburgh, PA). Foot-related responses were recorded through a foot-pedal response system connected with the computer running the E-Prime software.

Stimuli included a selection of pictures from a virtual reality environment, created using a 3D modeling software [3D Studio Max 4.2, Autodesk, Discreet, also see (Altomare et al., 2021; Di Marco et al., 2019; Tosoni et al., 2021)], representing a square arena of a three-winged palace with a landmark-object (e.g., a beach umbrella) positioned in front the central wing. For the current study, a series of pictures were created from the same 3D environment in which either the environmental layout, defined by the three-winged palace, or the landmark-object, defined by the beach umbrella, were framed from different (extrapersonal) distances from the observer (Fig. 1B). Extrapersonal distance was manipulated across trials by framing the environmental layout or the isolated landmark-object at 8 possible distances from the observer (5, 6, 7 and 8 virtual meters for the near condition and 28, 32, 36 and 40 virtual meters for the far condition). Specifically, as illustrated in Fig. 1B, the stimulation set employed in the current study was rendered by moving the camera at 8 possible distances (4 levels for each condition of near and far distance) along a vector connecting the camera with the landmark-object (beach umbrella) or with the three-winged palace in the arena [(note that pictures employed in the current study were rendered by simply removing the environmental layout or the landmark-object from the original stimulation set defined by the three-winged palace with the beach umbrella in front of the central wing described in (Di Marco et al., 2019)].

As in our previous studies (Altomare et al., 2021; Di Marco et al., 2019; Tosoni et al., 2021), distance was expressed in virtual meters (which have been estimated as approximately doubled with respect to real distances in the current environment) and the different levels of distance were selected based on the stimulation set and results obtained in our previous study on explicit judgments of extrapersonal distance in the same virtual environment [i.e., explicit categorization task of extrapersonal distance during ascending/descending series of receding/approaching distance of landmark-objects in the same environmental layout of the current study, see (Fini et al., 2014)]. In particular, conditions of near and far extrapersonal distance were selected to fall very far from the proposed ~ 8 m (i.e., 16 virtual) boundary defining a near- vs. far-distant extrapersonal action space (Fini et al., 2014; Grusser, 1983). It is worth specifying that the different conditions of extrapersonal distance employed in our study were expected to specifically modulate and differentially afford foot-related actions but not hand-related actions such as grasping or reaching.

#### Procedure

Experiment 1 was composed of two experimental sessions in which the spatial distance of the general environmental layout (i.e., square arena of a three-winged palace) or of an isolated object (i.e., a beach umbrella on the same arena without the three-winged palace) was manipulated across trials in a within-subject design. Within each session, a series of blocks were collected in which a footstep ahead or a simple release action was pre-instructed as a response movement.

As illustrated in Fig. 1C (C1 and C2 panels), the experimental design was based on a go/no-go incidental priming paradigm in which subjects were required to execute a previously instructed movement (simple release or footstep in separate experimental blocks) in response to repeated presentations of pictures (i.e., prime-target) of a 3D virtual environment representing a square arena of a three-winged palace (environmental layout session) or a beach umbrella (landmark-object session) positioned at different extrapersonal distances from the observer (near or far, 4 levels of distance each). The response was provided at the onset of the target picture (i.e., second picture) based on the perceptual match with the prime picture (i.e., go trials, execute the instructed response movement only when the target picture is perceptually identical to the prime picture; no-go trials, refrain the response when they are different).

During the experiments, subjects were instructed to respond by either simply releasing the foot-pedal with the heel of their right foot while keeping the foot’s front on the ground (simple release) or by releasing the pedal for executing a footstep ahead (footstep) and get back to the starting position (Fig. 1A). During the simple release action the leg remained still on the response pedal and the body weight loaded on the (left) non-responding foot, while during the footstep action the participants were instructed not only to move the leg for a step length but also the entire body ahead as if they were about to start walking. As a result, during the footstep action the body weight was shifted from the non-responding left to the responding right foot and this could be correctly executed without falling off the platform or running into the equipment. As reported in our original work (Di Marco et al., 2019), the footstep action was considered as a proxy of walking because of their shared basic postural, muscular and kinematic elements, but a simple release action was also included in the design as to control for task-related cognitive components (i.e., the perceptual difficulty component associated with the differential perceptual discriminability of experimental conditions in Experiment 1) as well as basic postural and proprioceptive aspects of the more complex footstep action. Therefore, in addition to the subtractive approach in which the simple release action was employed as a baseline/control for the footstep action, both response actions could be defined and interpreted as constitutive elements of a walking incipit (i.e., consecutive steps of a sensory-motor sequence for the initiation of a walking action).

Each picture was presented for 700 ms with an inter-stimulus interval (ISI) of 500 ms. An inter-trial interval (ITI) of 2500 ms was presented between every trial (pictures pair). Each session (layout, landmark-object) was composed of 4 blocks in which the instructed response movement was a simple release or a footstep action in a 2 (session) × 2 (response movement) × 4 (distance) within-subject factorial design. The response movement blocks were collected within a single session in a counterbalanced order across subjects (i.e., 4 consecutive blocks of simple release and footstep action in which the response movement was indicated before the beginning of the 4 blocks; half of the subjects performed the footstep blocks as first and the other half as second). The two sessions (layout, landmark-object) were collected in separate days to avoid dragging effects.

Each block was composed of 40 trials including 32 go trials (target picture equal to prime picture, 4 repetitions for each level of extrapersonal distance ranging from 5 to 40 virtual meters) and 8 no-go trials (target and prime picture of different distance categories) for a total of 640 trials collected for each participant (40 trials per block with a 1/5 ratio of no-go trials, 16 total blocks including 8 blocks per session, with each session including 4 blocks for each response movement). The response was provided at the onset of the target picture and the response time associated with the foot-pedal release was recorded on each trial. Before the beginning of the experiment, a brief training was performed to ensure that the two response movements were correctly executed (see above for a kinematic description of the response movements).

As reported above, go trials were defined by perceptually identical pictures within the near and far categories while no-go trials were defined by prime and target pictures extracted from different distance’ categories, i.e., near/far and vice versa, thus resulting in a very easy discrimination. More specifically, the very easy discrimination task employed in the current design satisfied the requirement of minimizing the potential contribution of task-difficulty modulations associated with differential perceptual discriminability of the distance conditions to performance (Di Marco et al., 2019). Therefore, by minimizing the contribution of task-related variables associated with stimulus–response mapping, the incidental priming paradigm was designed to emphasize modulations of implicit variables and automatic effects, such as an implicit coding of extrapersonal distance for the execution of a foot-related action. Alongside, the inclusion of no-go trials followed the rationale of controlling participants’ attention to visual stimulation and avoiding automatic and pre-programmed responses to target pictures.

#### Statistical analysis

The accuracy analysis was based on mean accuracy percentage in the go/no-go task (i.e., correct movement execution in go trials and correct movement withholding in no-go trials) for each subject and condition. Analysis of response time was conducted on the mean release times for correctly executed go trials (i.e., go trials in which a response movement was correctly executed) after removal of outliers trials (mean ± standard deviation of the mean calculated for each experimental block). Both accuracy and response time data were analyzed using a within-subject repeated-measures ANOVAs with session (layout, landmark-object), response movement (simple release, footstep), and distance (near, far) as factors.

The Newman–Keuls post hoc test was used for statistical comparisons between the mean scores in the different conditions.

### Results

A mean accuracy of 99% was recorded in the go/no-go task (i.e., go and no-go trials averaged) with no significant main effect or interaction in the ANOVA analysis (all *p* > 0.05). Therefore, together with our previous findings, these results suggest that the incidental priming paradigm developed for the study of the macro-affordance effect largely satisfies the experimental goal of controlling attention to the featured stimuli without compromising task performance (i.e., correct execution of the instructed response movement in the go/no-go task).

**Fig. 2:**
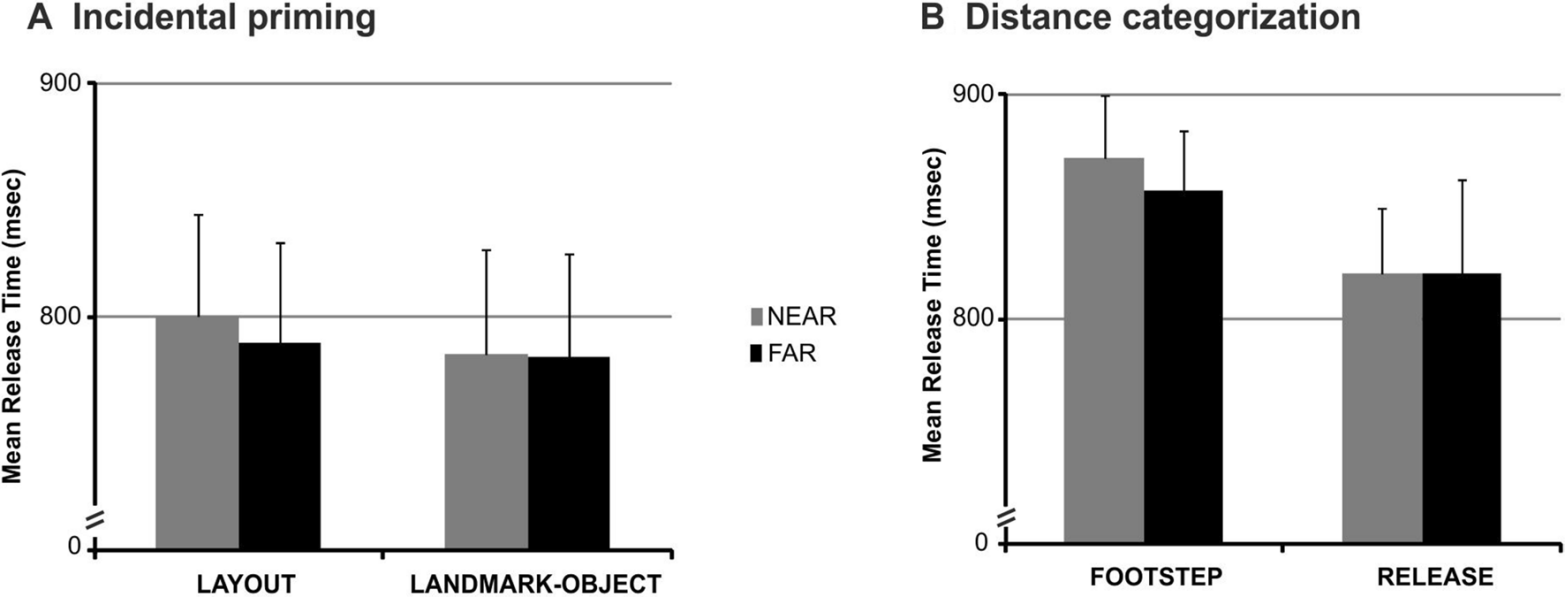
Experimental results. **A** Experiment 1: The graph displays the mean release times for the near and far conditions during the layout and landmark-object session of the incidental priming paradigm. Error bars represent within-subjects standard errors of the mean. **B** Experiment 2: the graph displays the mean release times for execution of the footstep and the simple release action as a function of distance (near, far) during the distance categorization task. Error bars represent within-subjects standard errors of the mean

The results of the response time ANOVA (i.e., mean release times for correctly executed go trials) indicated a main effect of distance [*F*(1,20) = 7.12, *p* = 0.01, partial eta-squared = 0.26, observed power (alpha 0.05) = 0.72] and a marginally significant session x distance interaction [*F*(1,20) = 3.47, *p* = 0.07, partial eta-squared = 0.15, observed power (alpha 0.05) = 0.43]. No other significant or marginally significant main effects or interactions were observed in the ANOVA results (all *p* > 0.07). As illustrated in Fig. 2A, whereas the main effect of distance was explained by overall shorter release times in the far vs. near condition (both simple release and footstep action), the marginally significant session x distance interaction suggested a larger distance effect in the layout vs. the landmark-object session (see also Table 1 for descriptive statistics). As reported above, no interaction effect was observed between session, distance and response movement, thereby indicating that the trend of a larger effect of distance in the layout vs. the landmark-object session was equally observed for the simple release and the footstep action. Notably, since the interaction effect did not reach the statistical significance of p < 0.05 but the observed trend was consistent with our hypothesis of a larger distance effect in the layout vs. the landmark-object session, post hoc tests were used to assess differences between the condition means. Consistent with our predictions, the results of post hoc tests indicated that the distance effect was present in the layout but not in the landmark-object session (Newman–Keuls post hoc test, near vs. far in layout: *p* = 0.005, in landmark-object: *p* = 0.62). Therefore, the current results indicated that the macro-affordance effect was preferentially guided by the framing distance of the environmental layout (far/wide/panoramic vs. near/close/restricted view) rather than of isolated landmark-objects (far vs. near) in the environment.

**Table 1:** Response times (mean ± standard deviation) for the ANOVA design of Experiment 1

	Footstep		Simple release	
	Far	Near	Far	Near
Layout	802.01 ± 187.42	815.96 ± 188.93	776.37 ± 235.55	784.75 ± 241.11
Object	786.09 ± 192.11	791.78 ± 191.67	778.73 ± 237.78	776.57 ± 232.26

## Experiment 2

Experiment 2 addressed the question of whether the macro-affordance effect was specifically observed in the context of an incidental priming paradigm as in Experiment 1 in which the distance dimension was elaborated at an implicit level or whether it could also be observed during a task requiring an explicit categorization of distance. To this aim, an explicit version of the go/no-go paradigm employed in Experiment 1 was developed in which the instructed response movements (simple release, footstep) were performed on the basis of an explicit distance categorization of the environmental layout (only pictures of the layout session were employed). A non-significant effect of distance in this explicit task would suggest that the macro-affordance effect is task-dependent and specifically related to an implicit coding of extrapersonal distance for the execution of foot-related actions. In contrast, a significant effect of distance during both the implicit and the explicit task would indicate that the effect is automatically activated independently from the task at hand.

### Methods

#### Participants

Twenty-four right-footed, healthy participants, recruited from students of the “G. D’Annunzio” University of Chieti Pescara (15 females; age range: 24.6 ± 4 years), were enrolled for experiment 2. Exclusion criteria were applied for pathological conditions and left-foot dominance. Sample size and sample selection criteria were the same as in Experiment 1.

#### Apparatus and stimuli

The experimental set-up and stimuli were the same used in Experiment 1.

#### Procedure

Experiment 2 employed the “layout” pictures of Experiment 1 (i.e., no landmark-object in the arena) in the context of a distance categorization task (Fig. 1D). The go/no-go task was implemented on single picture trials and subjects were requested to execute a previously instructed response movement (i.e., simple release, footstep) according to the perceived distance of the environmental layout (near, far). The stimulus–response association (i.e., the association between the response movement and the condition of distance) was alternated across blocks. For example, in a given block subjects were instructed to execute the footstep action when they judged that the environment was framed from a far/panoramic view and to refrain the response when judging that the environment was framed from a near/front view, while an opposite association was given in the subsequent block (i.e., executing the footstep action when the environment was framed from a near/front view and refraining the response when the environment was framed from a far/panoramic view).

Each picture was presented for 500 ms with an ITI of 2500 ms between each trial. The response was provided at the onset of each picture and the reaction time associated with the foot-pedal release was recorded on each trial. As for Experiment 1, the footstep and simple release actions were collected in separate blocks within a single session in a counterbalanced order across subjects (i.e., half of the subjects performed the footstep blocks as first and the other half as second).

A total of 8 blocks were collected in each participant (4 blocks for each response movement; 2 blocks for the go-far condition and 2 blocks for the go-near condition), each including 16 go trials and 16 no-go trials for a total of 256 trials per subject.

Before the beginning of the experiment, participants were extensively trained on both executions of the two response movements and the distance categorization task. In particular, a series of exemplars of the near (close view) and far (panoramic view) conditions was shown to participants and a brief training was conducted to control that the instructions were correctly understood and that the categorization task was accurately performed.

#### Statistical analysis

As for Experiment 1, accuracy analysis was based on mean accuracy percentage in the go/no-go task (go & no-go trials averaged) while response time analysis was based on the mean release times for correctly executed go trials. Accuracy and response time data were analyzed using a within-subject repeated-measures ANOVAs with response movement (simple release, footstep), and distance (near, far) as factors.

### Results

One subject was excluded from the analysis for an accuracy performance below 2 standard deviations from the mean. A mean accuracy of 96% was recorded for the remaining group (*N* = 23) with no significant differences between the conditions.

As displayed in Fig. 2B, the results of the response time ANOVA (i.e., mean release times for correctly executed go trials in the near/far) indicated a marginally significant main effect of response movement [*F*(1,22) = 3.95, *p* = 0.059, partial eta-squared = 0.15, observed power (alpha 0.05) = 0.47] with faster response time in the simple release vs. footstep condition, but no significant main effect of distance [*F*(1,22) = 0.18, *p* = 0.67] and no significant distance × response movement interaction [*F*(1,22) = 0.24, *p* = 0.62].

These results showed that the macro-affordance effect was specifically observed in the context of an incidental priming paradigm (Experiment 1) in which the distance dimension was implicitly processed, thereby suggesting a both automatic and task-dependent activation of macro-affordances.

## Experiment 3

Experiment 3 addressed the question of the relationship between the macro-affordance effect, in terms of response time modulation by framing distance of the environmental layout, and a specific response type/effector. In particular, by examining data of a manual version of the incidental priming paradigm employed in Experiment 1, the degree to which the macro-affordance effect is specifically associated with the execution of foot- vs. hand-related motor actions was examined in Experiment 3. At the level of the hypotheses, since a preferential affordance relationship was originally hypothesized between the distant extrapersonal environment and the locomotion action, no distance modulation was expected during the manual version of the incidental priming task. In addition, since the macro-affordance effect was interpreted on the basis of an automatic activation of mechanisms for spatial exploration/navigation of the environment in our previous studies (Altomare et al., 2021; Di Marco et al., 2019; Tosoni et al., 2021), a selectivity of the effect for the planning/execution of foot-related motor actions was hypothesized in the current experiment.

### Methods

#### Participants

Thirty right-footed, healthy participants, recruited from students of the “G. D’Annunzio” University of Chieti Pescara (9 females; age range: 22.2 ± 1.7 years), were enrolled for Experiment 3. Exclusion criteria were applied for pathological conditions and left-hand dominance.

#### Apparatus and stimuli

The experimental set-up and stimuli were the same used in the “layout” session of Experiment 1 with the only difference being that participants performed the experiment in a seated position in front of a desk with a manual response box.

#### Procedure

Experiment 3 only employed the “layout” pictures of Experiment 1 (i.e., no landmark-object in the arena) and was based on a manual version of the go/no-go incidental priming paradigm in which foot-related responses (simple release, footstep) were replaced with manual responses provided through a response button and performed by releasing the right index from the button press and executing a spatially directed hand-pointing movement in the stimulus direction. As for the foot-related version of the task, the response was provided at the onset of the target picture (i.e., go when the target picture is perceptually identical to the prime picture and refrain the response when pictures are different) and reaction times associated with the finger release were recorded on each trial. The manual version of the incidental priming paradigm was based on identical timing and stimulation trials/blocks of Experiment 1.

Before the beginning of the experiment, a brief training was performed on each participant to ensure that the task instructions were correctly understood and that the hand-pointing movement was accurately executed.

#### Statistical analysis

As for Experiments 1 and 2, accuracy analysis was based on mean accuracy percentage in the go/no-go task (go and no-go trials averaged) while response time analysis was based on the mean release times for correctly executed go trials. Mean accuracy and response times were calculated for each subject and condition (far, near) and compared through paired t-tests (Student T, two-tails, type I).

### Results

Analysis of the mean accuracy in the go/no-go task (mean percentage of correct responses in go and no-go trials) indicated an accuracy of 98% with no significant differences between the conditions [*t*(29) = 0.24, *p* = 0.80].

Consistent with our predictions, moreover, a no-significant effect of distance (i.e., near vs. far) was observed in the response time analysis of the manual version of the task [*t*(29) = 1.25, *p* = 0.22].

These results indicated that the far vs. near response time facilitation effect described for foot-related macro-affordances was not observed during a manual version of the incidental priming paradigm and suggested a foot-related specificity of the macro-affordance effect.

### Stimuli rating

In addition to the go/no-go tasks described above, an explicit rating was collected in the present study aimed at evaluating the locomotion-affording properties of the stimulation set employed in the laboratory experiments (Experiments 1–3).

### Methods

#### Participants

An independent sample of 95 participants (75 females, age range: 22.6 ± 6 years) was enrolled from students of the “G. D’Annunzio” University of Chieti Pescara for an explicit stimulus rating study conducted through an online survey.

#### Procedure

The stimulus rating study was conducted through an online questionnaire administered via the Qualtrics commercial platform.

During the questionnaire, participants were shown with 4 exemplar pictures of the experimental design (2 sessions × 2 distances, see Fig. 1B), and for each picture, a rating score was collected on the following conditions of viewer–environment interaction (i.e., affordance): (i) going for a walk and exploring the environment (spatial exploration); (ii) reaching of a specific object or spatial location (reaching of object/location): (iii) passively admiring the environment (passive admiration). The rating score was provided on a 7-point Likert scale. Specifically, a rating on a score ranging from 1 to 7 points was obtained for each of the 4 stimulus pictures on the following three questions: (i) express how much the picture pushes you to explore the environment via walking, (ii) express how much the picture pushes you to direct yourself toward a specific location of the environment, (iii) express how much the pictures pushes you to passively admire the environment.

#### Statistical analysis

Data of the stimulus rating experiment were firstly controlled for normal distribution using the goodness of fit Kolmogorov–Smirnov and Lilliefors tests for normal distribution.

Mean rating scores were next compared through repeated-measures ANOVAs with session (layout, object), distance (near, far) and condition of viewer–environment interaction (spatial exploration, reaching of object/location, passive admiration) as factors. The Newman–Keuls post hoc test was used for statistical comparisons between the mean scores in the different conditions.

### Results

Results of the normality check indicated a normal distribution of data (Kolmogorov–Smirnov D statistics ranging from 0.14 to 0.23 with *p* < 0.01 for all the 12 rating variables).

**Fig. 3:**
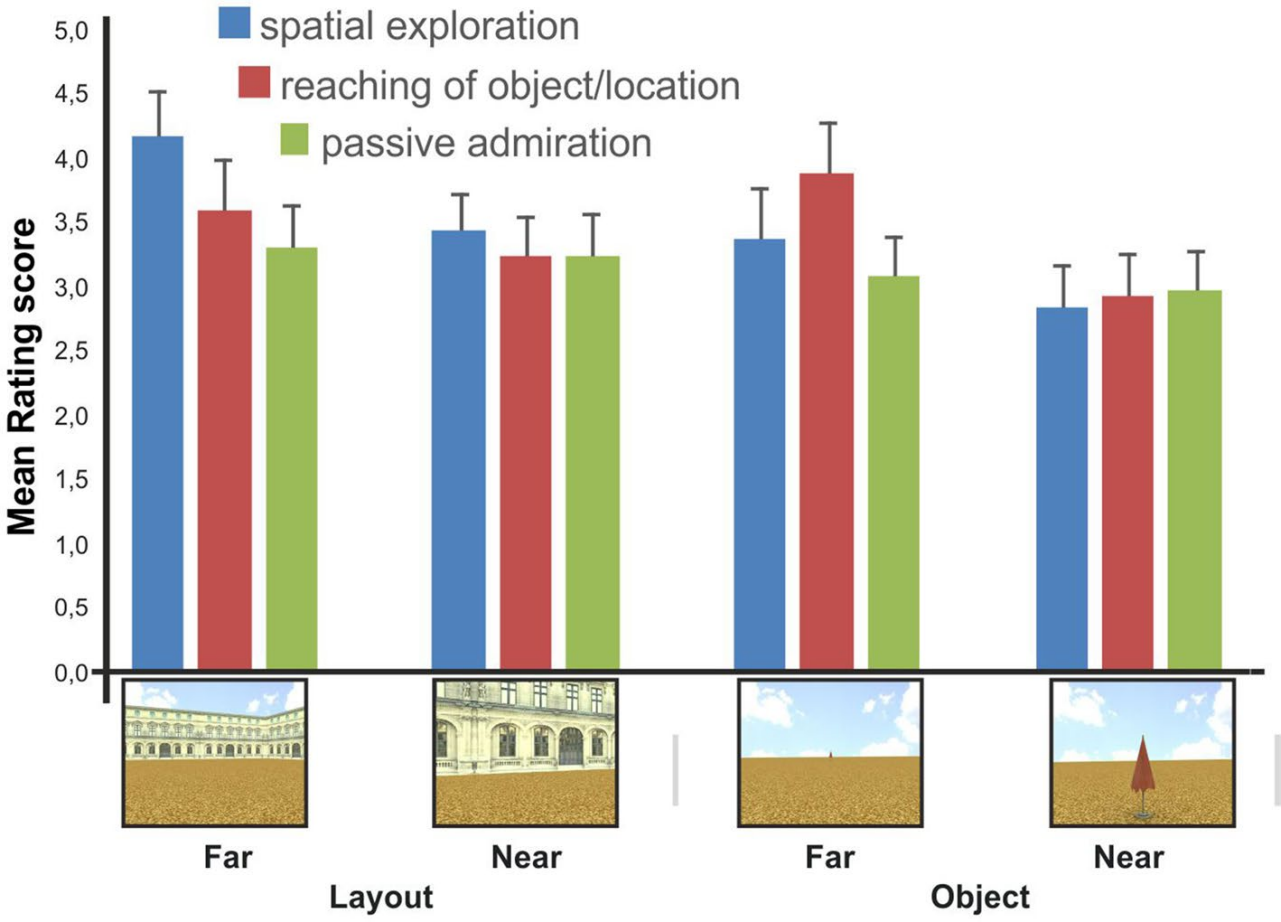
Rating results. The graph displays the mean rating score for the three conditions of viewer–environment interaction (i.e., spatial exploration, reaching of object/location, passive admiration) and stimulation condition [i.e., session (layout, landmark-object) by extrapersonal distance (near, far)]. Error bars represent within-subjects standard errors of the mean

As illustrated in Fig. 3, the mean rating scores associated with the three conditions of viewer–environment interaction indicated that the passive admiration condition obtained overall lower scores than the two “active” conditions of motor interaction with the environment, i.e., reaching of object/location and spatial exploration. This observation was statistically supported by the results of a within-subject repeated-measures ANOVA with session (layout, landmark-object), extrapersonal distance (near, far), and condition of viewer–environment interaction (spatial exploration, reaching of object/location, passive admiration) as factors. Specifically, a statistically significant three-way interaction was observed in the ANOVA [session x distance x condition of viewer–environment interaction: *F*(2,188) = 4.1, *p* = 0.018, partial eta-squared = 0.04, observed power (alpha 0.05) = 0.72] with post hoc tests (Newman–Keuls) indicating that the 4 admiration conditions obtained statistically comparable rating scores (*p* > 0.05) and that these scores were significantly lower than the rating scores in the 2 dominant “active” conditions of layout-far/exploration and landmark-object-far/reaching (all *p* < 0.05).

These results indicated that the 3D pictures presented in our laboratory experiments were strongly associated with an active, motor-related, interaction between the observer and the environment, thus confirming the strong locomotion-related affording properties of our stimulation set.

We next more specifically examined the potential dissociation between the two motor conditions of spatial exploration vs. landmark-object/location reaching by conducting a repeated-measures ANOVA with session (layout, landmark-object), distance (near, far) and condition of viewer–environment motor interaction (spatial exploration, reaching of object/location) as within-subject factors. Again, a significant three-way interaction between session, distance and motor condition [*F*(1,94) = 10.1, *p* = 0.002, eta-squared = 0.09, observed power (alpha 0.05) = 0.88] was observed in the ANOVA and the results of post hoc tests confirmed the dissociation between the preferred viewer–environment motor interaction and the type of stimulation condition (layout-far/exploration > layout-far/reaching, layout-near/exploration, landmark-object-far/reaching; landmark-object-far/reaching > layout-far/reaching, all *p* < 0.05).

Therefore, pictures of an environmental layout framed from a far/panoramic distance were associated with higher explicit judgments of spatial exploration than pictures of the same layout framed from a near/front distance and of a far isolated landmark-object. At the same time, pictures of a far landmark-object were associated with higher explicit judgments of reaching than pictures of a near landmark-object and of a near/far environmental layout. In this respect, note that the landmark-objects are conceived to represent environmental objects or “landmark” that afford a locomotion action (reaching in the sense of approaching) and not manipulable objects that afford a hand-related grasping action. Accordingly, the observed higher rating for “reaching” of object/location in the landmark-object-far vs. landmark-object-near condition closely followed the prediction of the “object reachability” hypothesis of the macro-affordance effect (i.e., when participants anticipate walking further their action starts faster).

In summary, the results of this explicit stimulus rating indicated that pictures of an environmental layout preferentially drive locomotion-related mechanisms of spatial exploration of the environment and suggest a preferential affordance relationship between the global environmental layout and the spatial exploration motor intention.

## General discussion

The brain has evolved to satisfy the request of a constantly changing environment that presents animals with multiple opportunities and demands for actions. As formalized by the classic notion of affordance (Gibson, 1979) and by the more recent neurophysiological model of the “affordance competition hypothesis” (Cisek, 2007; Cisek & Kalaska, 2010), one adaptive strategy to survive in such a complex environment is to specify in advance the parameters of all possible actions. Consistent with this view, a facilitation effect was described in our recent works for the execution of foot-related actions in response to distant vs. near objects/locations in the extrapersonal space (Altomare et al., 2021; Di Marco et al., 2019; Tosoni et al., 2021).

In the present study, we examined the degree to which this effect, which we have referred to as “macro-affordance” reflects an (i) implicit vs. controlled coding of (ii) environmental vs. landmark-objects’ distance for the planning/execution of (iii) specific response actions (e.g., foot- vs. hand-related motor actions).

The results indicate that the effect is preferentially guided by the framing distance of the environment layout (far/wide/panoramic vs. near/close/restricted view) rather than by the position of isolated objects in the environment (far vs. near) and that it only emerges when the distance dimension is processed at an implicit level (i.e., within the incidental priming paradigm).

The results additionally showed that the effect was specifically associated with execution of foot-related/walking (vs. hand-related) motor actions. Taken together, these findings suggest that the macro-affordance effect specifically reflects an implicit coding of environmental distance for the execution of foot-related actions that mimic the initiation of a walking.

Notably, the results of an explicit rating on the locomotion-related affording properties of our stimulation set complement these findings by indicating that far/panoramic views of the environmental layout preferentially elicit motor intentions and viewer–environment interactions associated with spatial exploration of the environmental layout.

These findings support our original interpretation of the macro-affordance effect in terms of an automatic trigger of walking by positions that preferentially guide spatial exploration (Di Marco et al., 2019). In particular, we propose that the observed preference of the distance effect for the “layout” session and the preferential association between pictures of the far/panoramic environmental layout and the “spatial exploration” interaction/intention support the hypothesis of a general and evolutionary-based mechanism associated with spatial exploration of the extended spatial environment.

The results presented in the current manuscript, however, also support the notion that the “object reachability” and the “spatial exploration” accounts can be thought of as two complementary, or at max hierarchically operating, mechanisms that together fulfill the purpose of adaptive interactions with the environment. Ultimately, also at the evolutionary level, when animals cover distance and reach distant (new) locations for food-hunting they probability also exploit the journey to explore the new environment for a different aim (e.g., colonize, settle down), thereby maximizing their survival chances.

An additional question addressed in the current study was the degree of automaticity of the macro-affordance effect and specifically whether the far vs. near distance facilitation effect observed during execution of foot-related actions was peculiarly associated with an implicit coding of environmental affordances or whether it was independent from the task at hand (Experiment 2). Albeit affordances, and in particular micro-affordances, have been classically considered as an automatic mechanism activated during object perception, mounting evidence from experimental psychology has indicated that affordances are not invariably activated during the presentation of pictures or words referring to manipulable objects: their activation is, therefore, both task- and context-dependent (Borghi & Riggio, 2015). For example, a series of studies have shown that affordance activation closely depends on the specific task employed in the study design and, in particular, that an affordance effect is observed during shape categorization but not during color categorization tasks (Pellicano et al., 2010; Tipper et al., 2006).

Here, we addressed the question of automaticity and contextual dependence of macro-affordances by comparing the modulation of performance by environmental distance during an incidental priming paradigm task in which the distance dimension was implicitly processed versus a task requiring an explicit categorization of the same dimension. The results indicated that the macro-affordance effect was significantly observed during the incidental priming task (Exp.1) but not during the distance categorization task (Exp. 2). Therefore, in accordance with previous experimental evidence and current theoretical views on affordance effects for manipulable objects, our results suggest that also affordance effects for environmental stimuli or macro-affordances are implicitly activated in a context-dependent manner.

A final question addressed in the present study is the degree to which macro-affordances are specifically associated with execution of foot-related/walking (i.e., footstep vs. simple release) and foot- vs. hand-related motor actions (Exp.3). In particular, although a specificity of the effect for the footstep action was observed in our original study (Di Marco et al., 2019), a modulation of distance for both the footstep and the simple release action was observed in the current study (i.e., larger effect of distance in the layout vs. the landmark-object session independently of the instructed response movement). In this respect, a considerable notation concerns the theoretical and methodological aspects of the experimental design. As reported in the methodological section of Experiment 1, in particular, although only the footstep action was taken as proxy of walking in our original work (Di Marco et al., 2019), also the simple release action was included in the design as control for basic aspect of the more complex footstep action. On this basis, therefore, a worthwhile theoretical consideration/specification is that both foot-related response actions can be thought of as constitutive elements of a walking incipit (i.e., consecutive steps of a sensory-motor sequence for the initiation of a walking action). At a more methodological level, moreover, it has to be noted that in the present study the two instructed movements were collected within the same session (see Experiment 1) which might have minimized the distinction/differentiation between the planning and execution components of the two response movements. Importantly, moreover, concerning the issue of the specificity of the macro-affordance effect for a particular response effector (foot- vs. hand-related motor actions), the results of an additional experiment indicated that the macro-affordance effect was not observed during a manual version of the incidental priming paradigm in which foot-related response responses were replaced with spatially directed hand-pointing movements. These findings suggested a foot-effector specificity of the macro-affordance effect.

On a more general level and with respect to previous empirical evidence, the findings presented in the current study are consistent with the findings of some follow-up studies in the field of micro-affordance showing that spatial alignment effects for simulated reach-to-grasp motor acts are computed online through behavior rather than reflected in explicit estimates or conscious representations of distance (Ambrosini & Costantini, 2013; Ambrosini et al., 2012). Here we found a similar pattern for the macro-affordance effect as directly estimated from a perceptual matching task in which the distance dimension was implicitly computed. This not only supports the conclusion of an online computation of affordance during task execution but also raises the hypothesis that the type of task might directly affect the relevance/salience of the distance dimension for action. More specifically, if one starts from the assumption that stimulus location is computed online for the purpose of the current behavioral goal, then one can formulate the hypothesis that the distance dimension is action-relevant for a foot-related response only during the implicit task. On the other hand, the distance categorization task might be primarily dominated by memory-recollection mechanisms associated with the memory encoding of the condition templates shown during the training phase of the task. According to our hypothesis, therefore, the role of perception (i.e., environmental distance) for action (foot-related action) would be largely preserved during the implicit task but rather impoverished by memory-recollection biases, and thus weekly available to mechanisms of perception for action, during the distance categorization task.

A further task-comparative aspect that might be worth discussing is the potential association between the observed macro-affordance effect and the incidental priming paradigm in which the effect was specifically observed. This is because the implicit but not the explicit distance categorization task was based on a priming paradigm and one might arise the concern that the observed facilitation effect might directly derive from a mechanism of pre-activation of the instructed response movement during the presentation of the prime stimulus. This potential priming explanation, however, would equally apply to the near and far conditions, for which we instead observed a differential modulation of the behavioral response. Therefore, the findings presented in the current study again support the notion that the macro-affordance effect particularly relies on the specific affording properties of the far condition, and in particular of the far and panoramic environmental condition, for execution of foot-related/walking actions.

Future studies, however, would hopefully further clarify the potential implication of the specific paradigm for the emergence of the macro-affordance effect and a set of questions that has remained unaddressed from the present and previous investigation on the “macro-affordance” effect.

One of these questions, for example, is the degree to which the perceptually constitutional aspects/features of the stimulation set employed in this study might affect the performance [see also (Witt et al., 2007) for relevant findings on this topic]. More specifically, a still unaddressed question is how the macro-affordance effect is modulated by the different ratio of depth cues (e.g., linear perspective, texture gradient) and stimulation of the visual field periphery in the two experimental conditions in which an isolated landmark-object or a distributed environmental layout was presented on the widescreen (also note that our studies were always conducted using the same 42″ widescreen covering 70° of visual angle).

An additionally relevant open question concerns the motivational basis of the effect and, in particular, whether within the evolutionary perspective outlined above, the macro-affordance effect can be conceived as associated with the activation of the so-called “seeking” disposition, a behavioral function of the mesolimbic dopaminergic system that is highly conserved during evolution and associated with exploratory and approaching behaviors such as forward locomotion (Panksepp, 2011; Panksepp & Biven, 2012). Within this broader perspective, in particular, we suggest that the proposed interpretation of the effect based on the unique role of locomotion in distance coverage and access to the information contained in distant extrapersonal locations is consistent with the definition of the seeking disposition in terms of “specific types of locomotor activities and other responses directed to attain perceptual information and to progressively orient the organism toward affectively enticing and eventually desired sources of stimulation” (Alcaro et al., 2007).

Finally, at a more general theoretical level, one relevant future direction would be to clarify the effect of automaticity and contextual dependence, which have been so far only addressed in the field of the affordance relationship between manipulable objects and upper limbs actions, i.e., micro-affordances, with specific reference to the distinction between stable and variable affordances [see (Borghi & Riggio, 2015) for a full discussion]. How the distinction between stable and variable affordances applies and translates to environmental affordances for lower limb actions, i.e., macro-affordances, remains still unexplored and requires future investigation.

In summary, future studies will hopefully further clarify the perceptual basis and the bottom-up factors driving the macro-affordance effect, its motivational significance and its contextualization in the affordance theory and ecological theories of perception (Richardson et al., 2008).

## Conclusion

Here, we showed that the facilitation effect observed in the current and previous studies for the execution of foot-related actions to environmental layouts framed from a far/wide/panoramic vs. near/close/restricted distance in the extrapersonal space (“macro-affordance”) is based on implicit coding of environmental affordances. At the interpretational level, based on the neurophysiological model of the “affordance competition hypothesis” (Cisek, 2007; Cisek & Kalaska, 2010) and the theorization of primary-process motivational systems, we propose that the macro-affordance effect might be thought of as a fingerprint of the evolutionary-based seeking disposition finalized to the exploration and navigation of our surrounding natural environment.

## References

[CR1] Alcaro A, Huber R, Panksepp J (2007). Behavioral functions of the mesolimbic dopaminergic system: An affective neuroethological perspective. Brain Research Reviews.

[CR2] Altomare EC, Committeri G, Di Matteo R, Capotosto P, Tosoni A (2021). Automatic coding of environmental distance for walking-related locomotion in the foot-related sensory-motor system: A TMS study on macro-affordances. Neuropsychologia.

[CR3] Ambrosini E, Costantini M (2013). Handles lost in non-reachable space. Experimental Brain Research.

[CR4] Ambrosini E, Scorolli C, Borghi AM, Costantini M (2012). Which body for embodied cognition? Affordance and language within actual and perceived reaching space. Consciousness and Cognition.

[CR5] Borghi AM, Riggio L (2015). Stable and variable affordances are both automatic and flexible. Frontiers in Human Neuroscience.

[CR6] Cisek P (2007). Cortical mechanisms of action selection: The affordance competition hypothesis. Philosophical Transactions of the Royal Society of London. Series B, Biological Sciences.

[CR7] Cisek P, Kalaska JF (2010). Neural mechanisms for interacting with a world full of action choices. Annual Review of Neuroscience.

[CR8] Costantini M, Ambrosini E, Scorolli C, Borghi AM (2011). When objects are close to me: Affordances in the peripersonal space. Psychonomic Bulletin & Review.

[CR9] Costantini M, Ambrosini E, Tieri G, Sinigaglia C, Committeri G (2010). Where does an object trigger an action? An investigation about affordances in space. Experimental Brain Research.

[CR10] Creem-Regehr SH, Kunz BR (2010). Perception and action. Wiley Interdisciplinary Reviews: Cognitive Science.

[CR345] Gibson, J.J. (1979). The ecological approach to visual perception. Houghton-Mifflin Co, Boston (MA), p 332.

[CR11] Di Marco S, Tosoni A, Altomare EC, Ferretti G, Perrucci MG, Committeri G (2019). Walking-related locomotion is facilitated by the perception of distant targets in the extrapersonal space. Science and Reports.

[CR12] Ellis R, Tucker M (2000). Micro-affordance: The potentiation of components of action by seen objects. British Journal of Psychology.

[CR13] Fini C, Costantini M, Committeri G (2014). Sharing space: The presence of other bodies extends the space judged as near. PLoS ONE.

[CR14] Grusser J, Jeannerod HM (1983). Multimodal structure of the extrapersonal space. Spatially oriented behavior.

[CR15] Panksepp J (2011). The basic emotional circuits of mammalian brains: Do animals have affective lives?. Neuroscience and Biobehavioral Reviews.

[CR16] Panksepp, J. & Biven, L. (2012). *The Archaeology of Mind: Neuroevolutionary Origins of Human Emotions*. New York.

[CR17] Pellicano A, Iani C, Borghi AM, Rubichi S, Nicoletti R (2010). Simon-like and functional affordance effects with tools: The effects of object perceptual discrimination and object action state. Q J Exp Psychol (hove).

[CR18] Richardson MJ, Shockley K, Fajen BR, Riley MA, Turvey MT, Gomila PCAT (2008). Ecological psychology: six principles for an embodied-embedded approach to behavior. Handbook of Cognitive Science: An Embodied Approach.

[CR19] Tipper SP, Paul MA, Hayes AE (2006). Vision-for-action: The effects of object property discrimination and action state on affordance compatibility effects. Psychonomic Bulletin & Review.

[CR20] Tosoni A, Altomare EC, Brunetti M, Croce P, Zappasodi F, Committeri G (2021). Sensory-motor modulations of EEG event-related potentials reflect walking-related macro-affordances. Brain Sciences.

[CR21] Tucker M, Ellis R (1998). On the relations between seen objects and components of potential actions. Journal of Experimental Psychology: Human Perception and Performance.

[CR22] Tucker M, Ellis R (2001). The potentiation of grasp types during visual object categorization. Visual Cognition.

[CR23] Witt JK, Stefanucci JK, Riener CR, Proffitt DR (2007). Seeing beyond the target: Environmental context affects distance perception. Perception.

